# In search of causal pathways in diabetes: a study using proteomics and genotyping data from a cross-sectional study

**DOI:** 10.1007/s00125-019-4960-8

**Published:** 2019-08-24

**Authors:** Kristina Beijer, Christoph Nowak, Johan Sundström, Johan Ärnlöv, Tove Fall, Lars Lind

**Affiliations:** 1 Department of Medical Sciences, Uppsala University, UCR, Dag Hammarskjölds väg 38, SE-751 83 Uppsala, Sweden; 2 Department of Neurobiology, Care Sciences and Society, Division of Family Medicine and Primary Care, Karolinska Institute, Stockholm, Sweden; 3 School of Health and Social Sciences, Dalarna University, Falun, Sweden

**Keywords:** Diabetes, Genotyping, Mendelian randomisation, Proteomics, Type 2 diabetes

## Abstract

**Aims/hypothesis:**

The pathogenesis of type 2 diabetes is not fully understood. We investigated whether circulating levels of preselected proteins were associated with the outcome ‘diabetes’ and whether these associations were causal.

**Methods:**

In 2467 individuals of the population-based, cross-sectional EpiHealth study (45–75 years, 50% women), 249 plasma proteins were analysed by the proximity extension assay technique. DNA was genotyped using the Illumina HumanCoreExome-12 v1.0 BeadChip. Diabetes was defined as taking glucose-lowering treatment or having a fasting plasma glucose of ≥7.0 mmol/l. The associations between proteins and diabetes were assessed using logistic regression. To investigate causal relationships between proteins and diabetes, a bidirectional two-sample Mendelian randomisation was performed based on large, genome-wide association studies belonging to the DIAGRAM and MAGIC consortia, and a genome-wide association study in the EpiHealth study.

**Results:**

Twenty-six proteins were positively associated with diabetes, including cathepsin D, retinal dehydrogenase 1, α-l-iduronidase, hydroxyacid oxidase 1 and galectin-4 (top five findings). Three proteins, lipoprotein lipase, IGF-binding protein 2 and paraoxonase 3 (PON-3), were inversely associated with diabetes. Fourteen of the proteins are novel discoveries. The Mendelian randomisation study did not disclose any significant causal effects between the proteins and diabetes in either direction that were consistent with the relationships found between the protein levels and diabetes.

**Conclusions/interpretation:**

The 29 proteins associated with diabetes are involved in several physiological pathways, but given the power of the study no causal link was identified for those proteins tested in Mendelian randomisation. Therefore, the identified proteins are likely to be biomarkers for type 2 diabetes, rather than representing causal pathways.

**Electronic supplementary material:**

The online version of this article (10.1007/s00125-019-4960-8) contains peer-reviewed but unedited supplementary material, which is available to authorised users.

## Introduction

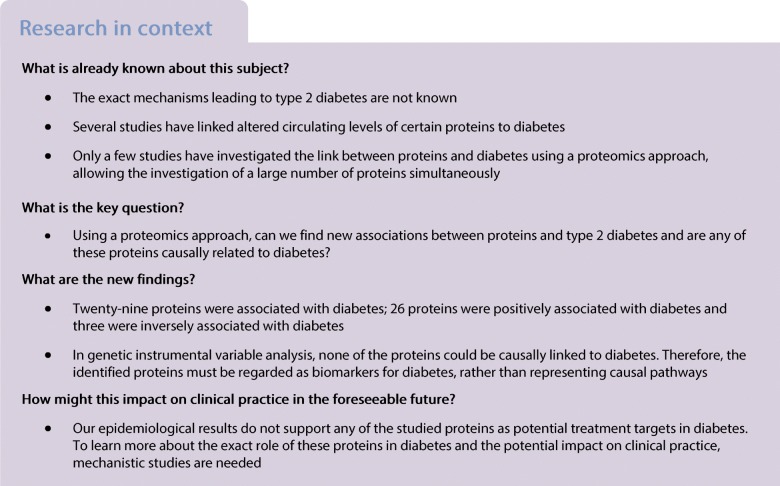



Although diabetes primarily could be seen as a disease in which the insulin secretion is not sufficient compared with insulin sensitivity in critical organs, the exact mechanisms leading to type 2 diabetes are not known. In the ‘-omics era’, several techniques have been used to search for the pathophysiological pathways underlying diabetes development, such as genome-wide association studies (GWASs) [1], DNA-methylation studies [2] and metabolomics studies [3, 4].

A number of studies have also linked alterations in certain proteins, such as C-reactive protein (CRP), γ-glutamyl transpeptidase (GGT) or adiponectin, to diabetes [5–8]. In recent years, a few studies used an antibody-based proteomics approach to link proteins to diabetes, which allows simultaneous investigation of multiple proteins [9–12]. One of these studies showed that plasma levels of IL-1 receptor antagonist (IL-1ra) and tissue plasminogen activator (t-PA) were related to incident diabetes [11]. Three other case–control studies of diabetes analysed proteins in urine by an untargeted approach using mass spectrometry. It was found that levels of histidine triad nucleotide-binding protein 1 (HINT1), bifunctional aminoacyl-tRNA synthetase (EPRS) and clusterin precursor protein (CLU) [9]; fibrinogen alpha chain precursor and prothrombin precursor [12]; and complement C3f and kininogen 1 isoform 1 precursor [10] were altered in individuals with diabetes.

We used the proximity extension assay technique to find proteins previously not known to be associated with diabetes. We evaluated 249 proteins and tested the hypothesis that a number of these would be related to prevalent diabetes in 2467 individuals in the general population-based EpiHealth study [13]. Using summary data from large GWASs for diabetes and related glycaemic traits and a GWAS of protein levels in the EpiHealth study, we performed a bidirectional two-sample Mendelian randomisation (MR) to test whether these proteins were causally related to diabetes, or whether diabetes could induce alterations in protein levels.

## Methods

### Sample

Between 2011 and 2016, men and women aged 45–75 years were randomly selected from the population registry in the Swedish cities Malmö and Uppsala and invited to participate in the cross-sectional EpiHealth cohort study [13]. The participation rate was approximately 20%. The recruiting and sampling of participants has been completed in Uppsala, but is ongoing in Malmö. A total of 2467 plasma samples from the Uppsala part of the EpiHealth cohort were randomly chosen for the protein and genotyping analyses. The study was approved by the regional ethical review board at Uppsala University (Dnr 2010/402). All participants provided informed, written consent.

### Examinations

BMI was calculated from the measured height and weight, as weight in kilograms divided by the square of body height in meters (kg/m^2^). Trained staff collected venous blood samples in the morning after a minimum 6 h fast at the EpiHealth test centre and stored them at −80°C. Diabetes was defined as either taking glucose-lowering treatment or having a fasting plasma glucose level of ≥7.0 mmol/l.

### Questionnaire

An extensive web-based questionnaire (EpiHealth Enkät version 1.0.1, available from https://www.epihealth.se/PageFiles/790/Enkät läskopia.pdf; in Swedish) was completed by the participants, including self-assessment of leisure time physical activity from low (level 1) to strenuous physical activity (level 5), sex, age, alcohol intake given as drinks per week, education length (up to 9 years, 10–12 years, >12 years) and tobacco use (current smoker, current non-smoker).

### Proteomic analysis

Analyses were performed at the Clinical Biomarkers Facility, Science for Life Laboratory, Uppsala University, with the high-throughput, multiplex immunoassays Olink Proseek Multiplex Metabolism, CVD II and CVD III (Olink, Uppsala, Sweden), measuring 275 preselected protein biomarkers of metabolism and cardiovascular disease (www.olink.com/products/document-download-center/; accessed October 2018). The kits are based on proximity extension assay technology, and in each kit 92 oligonucleotide-labelled antibody probe pairs can bind to their respective targets in the sample [14]. Correction for differences between plates was performed using ComBat (https://rdrr.io/bioc/sva/man/ComBat.html) in R (R Foundation for Statistical Computing, Vienna, Austria) [15]. Twenty-six proteins were removed from further analysis, since more than 25% of all samples were below the limit of detection (LOD).

The normalised protein concentrations (NPX-values) from the laboratory measurements (being on a log_2_ scale to achieve a normal distribution) were transformed to an SD scale to obtain comparable results for all proteins. Before the transformation to the SD scale, the values below the LOD were replaced by LOD/$\sqrt{2}$.

### Genotyping

Genotyping was performed for the same individuals for whom the protein analysis was done. Staff at the Biobank at Karolinska Institute extracted DNA from 400 μl EDTA whole blood with the Chemagen STAR DNA Blood 400 kit (Perkin Elmer, Waltham, MA, USA) using a ChemagicStar-robot (Hamilton, Reno, NV, USA) based on magnetic bead separation. DNA was dissolved in 145 μl 10 mmol/l Tris-HCl buffer (pH 8.0) and quantity and purity determined by measuring absorbance at 230, 260 and 280 nm. Subsequently, samples were genotyped at the SNP&SEQ Technology Platform, Science for Life Laboratory, Uppsala University with the Illumina HumanCoreExome-12 v1.0 BeadChip (Illumina, San Diego, CA, USA) including 522,731 autosomal markers.

The genotype data were initially called using Illumina GenomeStudio 2011.1 GenCall. Sample exclusion filters applied were: (1) samples with discordant sex information when comparing reported sex and sex determined by the X-chromosome; (2) outlying, non-European ancestry based on the first two components in a multidimensional scaling analysis (>3 SD from the mean); (3) outlying heterozygosity rate (>5 SD from the mean based on markers with a minor allele frequency [MAF] <1% or markers with MAF ≥1%); (4) low sample call rate (<98%); and (5) one individual in each pair of related individuals defined based on an identity-by-descent (IBD) analysis in PLINK [16] where a proportion >0.1875 was used as a cutoff for each pair. Markers with a call rate <97%, a Fisher’s exact test *p* value for Hardy–Weinberg equilibrium <10^−4^, a cluster separation score <0.4 or a GenTrain score <0.6 were also excluded. After rare variant genotype calling with zCall version 3.3 (https://github.com/jigold/zCall), markers with a call rate <99% or a Fisher’s exact test *p* value for Hardy–Weinberg equilibrium <10^−4^ were also excluded. Further details can be found in the study by Kamble et al [17]. In total, 2432 samples passed quality control, and 2378 samples remained after further exclusion of related individuals. Data were imputed up to 1000 Genomes phase 3 (v5) (http://www.internationalgenome.org/) and the final genetic dataset included approximately 12 million markers (minor allele count ≥1).

### Statistical analysis

#### Observational study (protein levels vs diabetes)

A discovery/validation approach was applied in that a random subset of two-thirds of the sample was used in the discovery step and the remaining one-third of the sample was used for validation. The level of significance was set to a false discovery rate (FDR) of 5% in both discovery and replication analyses.

A series of logistic regression models was applied to assess the association of each protein with diabetes. Adjustment was performed for age, sex, BMI, smoking, alcohol intake, education level and leisure time physical activity.

Stata 14 (Stata, College Station, TX, USA) was used for these calculations.

A power analysis was performed for the MR analysis (electronic supplementary material [ESM] Table 1) using free software (https://sb452.shinyapps.io/power/). The *R*^2^ is the protein level variance explained by the lead SNP. The power for the MR analysis was calculated with this free software for an OR of 1.1 (or 0.9) using the Diabetic Genetics Replication and Meta-analysis consortium (DIAGRAM) study by Xue et al [18], with 62,892 type 2 diabetes cases and 596,424 control individuals and a significance level of *p* = 0.05.

### Two-sample, bidirectional MR analyses

We implemented bidirectional instrumental variable analysis to assess causality between proteins observationally associated with diabetes and insulin resistance (measured by HOMA-IR), fasting glucose and risk of type 2 diabetes. We obtained summary GWAS data from the Meta-Analysis of Glucose and Insulin-related traits Consortium (MAGIC), Dupuis et al [19], for HOMA-IR (up to 46,186 non-diabetic individuals), from Scott et al [20] for fasting glucose (up to 108,557 individuals) and from the DIAGRAM consortium, Xue et al [18], for risk of type 2 diabetes (62,892 case and 596,424 control individuals). All three studies meta-analysed GWASs in mostly European participants with adjustments for sex, age and genetic principal components.

To select genetic instruments for type 2 diabetes and fasting glucose, we selected all SNPs associated at *p* < 5 × 10^−8^ and used the *clump_data()* function in the TwoSampleMR software package in R to prune SNPs in linkage disequilibrium (LD) with the default clumping window of 10,000 kb and *r*^2^ > 0.001. There were no SNPs associated at *p* < 5 × 10^−8^ with HOMA-IR, and we therefore extracted summary data for ten SNPs previously validated as part of a genetic risk score for insulin resistance by Scott et al [21]. We then extracted details of SNP associations with protein levels from the EpiHealth GWAS using proxy SNPs in LD *r*^2^ > 0.7 for SNPs not available in EpiHealth. Proxy search was carried out in LDLink using the European reference population (https://ldlink.nci.nih.gov). SNPs were aligned to the effect allele using the *harmonize_data()* function with the recommended option *action = 2* which harmonises SNPs by trying to infer forward strand alleles using allele frequency information, and excludes palindromic, non-inferable SNPs. The inverse variance-weighted and MR Egger methods were used to estimate instrumental variable effects. Heterogeneity and horizontal pleiotropy were assessed by the Q statistic and Egger intercept term at the nominal significance level. We consider as results statistical evidence of causal effects in the inverse variance-weighted method (Bonferroni corrected for the number of tested proteins and three outcomes per protein) with directionally consistent estimates in MR Egger and no statistical evidence of heterogeneity (Q statistic) and horizontal pleiotropy (Egger intercept).

In order to assess causal effects of proteins on the three phenotypes, we carried out *cis*-MR by constricting the selection of genetic instruments for protein levels to genome-wide-associated variants within each protein’s gene locus ±1000 base pairs. By restricting selection to SNPs within the protein locus, *cis*-MR minimises the likelihood of pleiotropic effects not mediated by the protein of interest [22]. To identify *cis* genetic instruments for observationally diabetes-associated proteins, we extracted all SNPs associated at *p* < 5 × 10^−8^ with protein levels and located within the gene locus in the EpiHealth GWAS (ESM Table 2). If several signals were identified, LD pruning was implemented as described above. We also searched the GWAS results (1) from Sun et al [23], who identified 1927 independent genetic associations with 1478 proteins out of ~3600 tested proteins in a sample of 3301 European participants; (2) from Suhre et al [24] (available via http://metabolomics.helmholtz-muenchen.de/pgwas/index.php), who carried out a GWAS for 1124 proteins in 1000 German and 338 Arab individuals; and (3) collected in the GWAS catalogue (https://www.ebi.ac.uk/gwas/). SNP associations with the outcomes type 2 diabetes risk, fasting glucose and HOMA-IR were extracted from the three meta-GWASs [18–20]. Instrumental variable analysis was implemented as described above for multiple-SNP instruments or using the Wald ratio and delta method to estimate the SE in cases where a single SNP was selected as instrument, as implemented with default options by the *mr()* function in TwoSampleMR [25].

## Results

In total, 211 (8.5%) individuals had prevalent diabetes. Basic characteristics in the discovery and validation subsamples are given in Table 1.

**Table 1:** Basic characteristics in the discovery and validation subsamples of the observational study in EpiHealth

Variable	Discovery subsample (*n* = 1645)	Validation subsample (*n* = 822)
Age (years)	60.4 (8.3)	61.0 (8.4)
Women (%)	49	52
BMI (kg/m^2^)	26.5 (3.8)	26.5 (3.9)
Fasting plasma glucose (mmol/l)	6.0 (0.9)	6.0 (1.0)
Diabetes (%)	8.4	8.8
Years in education (%)		
<10	21	23
10–12	28	30
>12	51	47
Years of smoking	8.7 (9.1)	9.0 (9.3)
Drinks a week	2.4 (3.0)	2.3 (2.5)
Physical activity (scale^a^ 1–5)	3.0 (0.9)	3.0 (0.9)

Data are displayed as mean (SD) unless indicated otherwise

^a^Physical activity scale from low (1) to high (5)

### Observational study (proteins vs diabetes)

In the discovery part of the study, 68 proteins were associated with diabetes at an FDR of 5%. Of these, 29 could be validated at an FDR of 5%. An increase in the levels of the following proteins was associated with higher odds of having diabetes (top ten findings): cathepsin D (CTSD), retinal dehydrogenase 1, alpha-l-iduronidase (IDUA), hydroxyacid oxidase 1 (HAO1), galectin-4 (GAL-4), growth/differentiation factor 15 (GDF-15), IL-1ra protein, cathepsin O (CTSO), sialic acid-binding Ig-like lectin 7 and plasminogen activator inhibitor 1. Only three proteins were associated with lower odds of diabetes, i.e. lipoprotein lipase (LPL), IGF-binding protein 2 and paraoxonase 3 (PON-3) (Table 2).

**Table 2:** OR, 95% CI and *p* value for the 29 proteins associated with prevalent diabetes in the validation analysis of the observational study in EpiHealth

Protein	Short name	Main function	Previously associated with diabetes (PMID)^a^	OR (95% CI)	*p* value
Cathepsin D	CTSD	Protein degradation	30670722 HR 1.33 (1.13, 1.56)	1.79 (1.38, 2.32)	9.56 × 10^−6^
Retinal dehydrogenase 1	ALDH1A1	Formation of retinoic acid	24464599 + *p* < 0.05	1.71 (1.34, 2.19)	2.09 × 10^−5^
α-l-iduronidase	IDUA	Hydrolysis of dermatan sulfate		1.92 (1.36, 2.71)	1.88 × 10^−4^
Hydroxyacid oxidase 1	HAO1	2-hydroxyacid oxidase		1.69 (1.28, 2.24)	2.56 × 10^−4^
Galectin-4	Gal-4	Modulating cell-matrix interactions	30670722 HR 1.37 (1.15, 1.64)	1.69 (1.28, 2.25)	2.58 × 10^−4^
Growth/differentiation factor 15	GDF-15	Regulating inflammation and apoptosis	22997280 + *p* < 0.0001	1.77 (1.3, 2.41)	2.78 × 10^−4^
Lipoprotein lipase	LPL	Hydrolysis of triacylglycerols	30326043 OR 0.69 (0.62, 0.76)	0.55 (0.4, 0.77)	5.33 × 10^−4^
IL-1 receptor antagonist protein	IL-1ra	Immune and inflammatory responses	26420861 HR 1.28 (1.03, 1.59)	1.75 (1.27, 2.42)	6.64 × 10^−4^
Cathepsin O	CTSO	Protein degradation		1.61 (1.22, 2.12)	7.47 × 10^−4^
Sialic acid-binding Ig-like lectin 7	SIGLEC7	Sialic acid dependent binding to cells		1.64 (1.22, 2.21)	0.0011
Plasminogen activator inhibitor 1	PAI-1	Involved in fibrinolysis	30670722 HR 1.70 (1.41, 2.05)	1.65 (1.22, 2.24)	0.0013
C-C motif chemokine 16	CCL16	Chemoattractive for monocytes and lymphocytes	28840653 + *p* = 0.04	1.91 (1.28, 2.84)	0.0014
E-selectin	SELE	Cell surface adhesion protein	30463448 + *p* = 0.008	1.71 (1.23, 2.37)	0.0015
Cathepsin Z	CTSZ	Protein degradation		1.63 (1.2, 2.23)	0.0019
ACE 2	ACE2	Formation of angiotensin		1.48 (1.15, 1.91)	0.0024
V-set and immunoglobulin domain-containing protein 2	VSIG2	Unknown		1.55 (1.16, 2.07)	0.0027
Ectonucleotide pyrophosphatase/phosphodiesterase 7	ENPP7	Converts sphingomyelin to ceramide		1.52 (1.16, 2.01)	0.0028
Tartrate-resistant acid phosphatase type 5	TR-AP	Glycosylated metalloprotein enzyme		1.62 (1.18, 2.22)	0.0031
Cadherin-2	CDH2	Cell adhesion protein		1.56 (1.16, 2.11)	0.0034
IGF-binding protein 2	IGFBP-2	Inhibits IGF-mediated growth	30670722 HR 0.66 (0.56, 0.77)	0.64 (0.47, 0.88)	0.0052
Fatty acid-binding protein, adipocyte	FABP4	Carrier protein for fatty acids	30670722 HR 1.74 (1.44, 2.10)	1.69 (1.17, 2.46)	0.0055
Aromatic-l-amino-acid decarboxylase	DDC	Decarboxylation of amino acids		1.51 (1.12, 2.04)	0.0067
Paraoxonase (PON 3)	PON3	Hydrolyses lactones andbinds to HDL	30670722 HR 0.65 (0.56, 0.75)	0.66 (0.49, 0.9)	0.0080
IL-1 receptor type 1	IL-1RT1	Inflammation		1.48 (1.11, 1.98)	0.0085
Tissue plasminogen activator	t-PA	Involved in fibrinolysis	26420861 HR 1.30 (1.03, 1.65)	1.63 (1.13, 2.35)	0.0088
T-cell and immunoglobulin and mucin domain-1/kidney injury molecule 1	TIM-1/KIM-1	T helper cell development	24904085 + *p* < 0.001	1.5 (1.09, 2.06)	0.013
Protease serine S1 family member 8	PRSS8	Serine protease		1.52 (1.07, 2.14)	0.018
P-selectin glycoprotein ligand 1	PSGL-1	Adhesion molecule		1.49 (1.07, 2.07)	0.019
Nodal modulator 1	NOMO1	Antagonise nodal signalling		1.4 (1.05, 1.88)	0.024

^a^Details of whether these proteins have previously been linked to human diabetes, giving the PMID number to that publication and strength (or direction) of the association. Only the proteins with an FDR <5% in the validation step were included in the Table. OR/HR and 95% CI are given, or direction and *p* value

As indicated in Table 2, only 15 of the 29 identified proteins are known to be linked to human diabetes. The remaining 14 are novel protein associations.

The majority of these 29 validated proteins were correlated, with Pearson’s *r* ranging from −0.30 to 0.51, as apparent in the heatmap in ESM Fig. 1.

In an additional analysis excluding the 64 study participants using glucose-lowering drugs, the ORs for most of the 29 proteins found significant in the main analysis were shifted towards 1, indicating weakened associations with diabetes (ESM Table 3). Major shifts in the ORs in this additional analysis were seen for GAL-4, GDF-15, CTSO, T-cell and immunoglobulin and mucin domain-1 (TIM-1; also known as kidney injury molecule 1 [KIM-1]) and nodal modulator 1 (NOMO-1).

The participants taking glucose-lowering drugs showed a similar age, sex-distribution and BMI to the participants with diabetes not taking glucose-lowering drugs, but the fasting glucose level was significantly higher in the participants on glucose-lowering drugs (8.9 vs 7.7 mmol/l, *p* < 0.001).

In an analysis with additional adjustment for glucose-lowering medication (but keeping those with glucose-lowering medication in the sample), 16 of the proteins showed FDR <5% in the validation step (see ESM Table 2 for details).

### Mendelian randomisation

#### Effect of protein levels on type 2 diabetes, fasting glucose and HOMA-IR

We identified *cis*-acting genetic instrumental variables in the EpiHealth GWAS for ten proteins (C-C motif chemokine 16 [CCL16], CTSD, IDUA, IL-1ra, LPL, TIM-1, V-set and immunoglobulin domain-containing protein 2 [VSIG-2], GDF-15, IL1-R1, PON-3), but some of the variants (or proxies with *r*^2^ > 0.7) were not available in GWAS results for some of the outcomes. We additionally identified *cis*-acting instrumental variables for seven proteins (IDUA, IL1-ra, TIM-1, GDF-15, ectonucleotide pyrophosphatase/phosphodiesterase 7 [ENPP-7], selectin P ligand [SELPLG] and CTSD) by searching previous GWAS repositories and results reported by Sun et al [23] (ESM Table 4). The median variance explained (*R*^2^) for the levels of these proteins was 0.054. MR results for effects of proteins on glycaemic traits are given in ESM Table 5.

Genetically raised LPL levels were associated with increased risk of type 2 diabetes (OR per SD unit increase, 1.10; 95% CI 1.06, 1.15; *p* = 6.7 × 10^−7^), with a directionally consistent but statistically weak effect on insulin resistance (change in natural log-scaled HOMA-IR per SD unit, 0.02; 95% CI 0.00, 0.04; *p* = 0.046, ESM Table 5). The underlying instrument for this analysis was the *LPL* SNP rs325 (effect allele C, MAF 13.0%, here decreasing LPL), which is in complete LD with the well-described *LPL* gain-of-function premature stop codon-inducing variant rs328 (effect allele G, MAF 13.0%). Given that the G-allele of rs328 is well known to cause increased levels and activity of LPL [26], probably through decreased translational inhibition [27], we question the validity of the association of the C-allele with decreased levels of LPL in our data. We speculate that the premature stop codon introduced by the G-allele may affect antibody affinity of our assay and thereby interfere with accurate measurement of LPL levels in carriers. The association of the G-allele of rs328 with lower triacylglycerols, lower risk of diabetes and increased insulin resistance is well-documented in the literature [28–31] (available on searching ‘rs328’ at http://www.phenoscanner.medschl.cam.ac.uk/). To further explore this issue, we re-assessed LPL using another instrument, the rs6999612, in low LD (0.001) with the gain-of-function SNP rs328. The T-allele of this SNP was associated with increased LPL in EpiHealth (β 1.9, SE 0.17, *p* = 2.2 × 10^−29^) and provided a causal estimate of OR 0.970 (0.922, 1.020) for type 2 diabetes.

#### Effect of diabetes, fasting glucose and insulin resistance on protein levels

We selected 120 SNPs for type 2 diabetes, 35 SNPs for fasting glucose and ten SNPs for HOMA-IR as genetic instrumental variables. The final set was reduced to nine SNPs for HOMA-IR after removal of one SNP with no available proxy (rs731839), and 29 SNPs for fasting glucose after removal of six SNPs with ambiguous allele harmonisation. We adjusted analyses for multiple testing (*p* < 0.05/29 proteins × 3 outcomes). The results from the glucose trait to protein part are given in ESM Table 6. Genetically raised fasting glucose was associated with reduced levels of fatty acid-binding protein 4 (FABP4) (level change in SD unit per mmol/l increase in fasting glucose, −0.76; 95% CI −1.18, −0.35; *p* = 3.3 × 10^−4^), with directionally consistent estimates in MR Egger and no evidence of heterogeneity (*p* > 0.05). A risk-decreasing effect on type 2 diabetes of raised PON-3 levels using the inverse variance-weighted method (*p* = 3.0 × 10^4^) was cast in doubt by evidence of heterogeneity (*p* = 3.1 × 10^−2^) and horizontal pleiotropy in MR Egger (*p* = 0.033). A decreasing effect on HOMA-IR by raised PON-3 levels was found in inverse variance-weighted MR (*p* = 2.5 × 10^−5^), with directionally consistent estimates in MR Egger and no evidence of heterogeneity (ESM Table 6).

## Discussion

The present study identified 29 proteins being associated with prevalent diabetes. Of these, 14 had not previously been described to be linked with human diabetes. The MR part of the study did not, however, disclose any significant causal effects of the proteins on diabetes, or of diabetes on the proteins, that were consistent with the relationships found between the protein levels and diabetes.

### Comparison with the literature

Approximately half of the identified proteins being linked to diabetes in the present study were not previously known. Amongst the top ranked new associations were IDUA and HAO1, representing two biological pathways of possible interest for diabetes: breakdown of glycosaminoglycans and 2-hydroxyacid oxidase activity.

The present study also confirmed some other previously published protein vs diabetes relationships. Amongst our top findings, CTSD, retinal dehydrogenase 1 and galectin-4 were such validations of previously published associations.

### Mendelian randomisation

By use of MR, we could not show any causal effects that were concordant with relationships found for protein levels vs prevalent diabetes.

A poor activity of the enzyme LPL has previously been linked to insulin resistance [32] and diabetes [33], in accordance with our finding of a negative association between LPL concentrations and prevalent diabetes. In addition, in a large study of genetic variants of the LPL gene associated with lowering of triacylglycerols, a positive association with diabetes was found, giving genetic evidence of a benefit of high LPL activity [28]. In the MR part of our study, however, a positive association between genetically determined LPL concentration and diabetes was found. This was probably due to the fact that as genetic instrument we had used a SNP in complete LD with an *LPL* gain-of-function premature stop codon-inducing variant, which possibly had changed the affinity of the antibody used for LPL measurements.

We mainly used a GWAS study based on the almost 2500 individuals in the present study. It might have been that this number was too small to detect a significant causal effect. However, the power analysis presented in ESM Table 1 showed that we would have a good power to detect causal estimates corresponding to ORs below 0.9 and above 1.1 for most of the instrumental variables used.

In a recent systematic overview of biomarkers for diabetes, the authors investigated 139 articles describing over 372 biomarkers, and identified 167 unique biomarkers, which had been evaluated at least once [34]. Between 53% and 76% of these biomarkers showed significant associations with type 2 diabetes, depending on the sample size of the study. The authors also evaluated the literature regarding the biomarkers that have been evaluated by MR, and showed that for ferritin [35], N-terminal pro brain natriuretic peptide (NT-proBNP) [36] and resistin [37] there is published evidence of a causal role for these proteins regarding diabetes. The finding for NT-proBNP is in contrast to the present study, since NT-proBNP levels were not associated with diabetes. Such a discrepant finding could be due to differences in sample characteristics or assays used.

In the additional analysis, excluding the individuals taking glucose-lowering medication, we noted as expected a tendency for a deviation of the ORs towards 1.0 for most proteins, since we have excluded those with the longest history (and possibly the worst severity) of diabetes. However, we noticed a substantial deviation of the ORs towards 1.0 for some of the proteins, such as GDF-15, which might be due to reverse causation, in this case an effect of the glucose-lowering medication on the protein in the main analysis. Also, an additional adjustment for glucose-lowering treatment in the main analysis would reduce the impact of glucose control, since individuals on glucose-lowering medication showed a higher fasting glucose level than individuals with diabetes without treatment.

### Strengths and limitations

A strength of the present study is the high number of proteins evaluated within a fairly large population-based sample. Another strength is that we could perform a bidirectional two-sample MR, since both proteomics and genotyping data exist in the EpiHealth study. The major limitation is the cross-sectional nature of the study. Therefore, the present results must be confirmed in future prospective studies with incident cases of diabetes. Another limitation is the lack of replication in an independent sample. We therefore used the second-best approach, namely the split sample approach, and performed the discovery/validation procedures within the sample. It should also be acknowledged that the sample consists of people of European ancestry, and therefore the generalisability might be limited for other ethnic groups.

In this study, we used a definition of diabetes based on a previous diagnosis or one measurement of fasting glucose level ≥7 mmol/l. In the clinic, two measurements are usually warranted for a diabetes diagnosis, but it is very uncommon to have two measurements in epidemiological studies. Therefore, it is likely that a slight overestimation of the prevalence of diabetes has occurred in the present study. However, a misclassification of non-diabetic individuals to individuals with diabetes would only drive the results towards the null hypothesis and would not cause any false positive results.

## Conclusion

The 29 proteins associated with diabetes are involved in several physiological pathways, but, given the power of the study, no causal link was identified for those proteins tested in MR. Therefore, the identified proteins are likely to be biomarkers for diabetes, not likely representing causal pathways.

## Electronic supplementary material

ESM (PDF 295 kb)

## Data Availability

The datasets generated during and/or analysed during the current study are not publicly available due to lack of informed consent from the participants to share data publicly, but are available from the corresponding author on reasonable request.

## Abbreviations

CTSD: Cathepsin D
CTSO: Cathepsin O
DIAGRAM: Diabetic Genetics Replication and Meta-analysis
FDR: False discovery rate
GAL-4: Galectin-4
GDF-15: Growth/differentiation factor 15
GWAS: Genome-wide association study
HAO1: Hydroxyacid oxidase 1
IDUA: α-l-iduronidase
IL-1ra: IL-1 receptor antagonist
LD: Linkage disequilibrium
LOD: Limit of detection
LPL: Lipoprotein lipase
MAF: Minor allele frequency
MAGIC: Meta-Analysis of Glucose and Insulin-related traits Consortium
MR: Mendelian randomisation
NT-proBNP: N-terminal-pro brain natriuretic peptide
PON-3: Paraoxonase 3
TIM-1: T-cell and immunoglobulin and mucin domain-1 (also called KIM-1, kidney injury molecule 1)
