# Supplementary material for: In search of causal pathways in diabetes: a study using proteomics and genotyping data from a cross-sectional study
Source: Diabetologia. 2019 Aug 24;62(11):1998–2006. doi: 10.1007/s00125-019-4960-8 (PMC6805963; doi:10.1007/s00125-019-4960-8)

ESM Table 1. Power calculation for the MR analysis. The R^2 is the protein level variance explained by the lead SNP. The power for the MR-analysis was calculated by the free software (https://sb452.shinyapps.io/power/) for an odds ratio of 1.1 (or 0.9) using the study by Xue et al (21) with 62,892 T2D cases and 596,424 control persons and a significance level of p=0.05.

Gene	SNP rs-number	R^2	Power
**EpiHealth GWAS (SNP-exposure associations)**			
CCL16	rs11080369	0.205	100
CTSD	rs7122341	0.046	100
IDUA	rs3796622	0.291	100
IL-1Ra	rs4251961	0.027	96
LPL	rs325	0.054	100
TIM-1	rs6878069	0.213	100
VSIG2	rs11219769	0.029	97
GDF15	rs1227731	0.052	100
IL1RT1	rs1024794	0.0192	89
PON3	rs10487132	0.062	100
**Previously published GWAS (SNP-exposure associations)**			
CTSD	rs55861089	0.054	100
SELPLG	rs73191242	0.013	75
IDUA	rs3822020	0.220	100
CATZ	rs199825459	0.092	100

ENPP7	rs11871061	0.426	100
GDF15	rs45543339	0.102	100
IL-1Ra	rs6761276	0.027	96
TIM-1	rs6889164	0.059	100
Siglec-7	rs140185670	0.043	100

ESM Table 2. Odds ratio (OR), 95% confidence interval (CI) and p-value for the 29 proteins associated with prevalent diabetes in the validation analysis of the observational study in EpiHealth following adjustment for diabetes medication. Only the proteins with a false discovery rate
(FDR) <5% in the validation step were included in the table.

Protein	Short name	OR	Lower 95%CI limit	Higher 95%CI limit	p-value
Cathepsin D	CTSD	1.79	1.38	2.32	9.56e-06
Hydroxyacid oxidase 1	HAOX1	1.69	1.28	2.24	0.0002564
Galectin-4	Gal-4	1.69	1.28	2.25	0.0002575
Lipoprotein lipase	LPL	0.55	0.4	0.77	0.0005328
Interleukin-1 receptor	IL-1ra	1.75	1.27	2.42	0.0006643

antagonist protein					
Gastric intrinsic factor	GIF	1.73	1.26	2.38	0.000752
Sialic acid-binding Ig-like lectin 7	SIGLEC7	1.64	1.22	2.21	0.0011108
Plasminogen activator inhibitor 1	PAI	1.65	1.22	2.24	0.0013094
Cathepsin Z	CTSZ	1.63	1.2	2.23	0.0018956
Cadherin-2	CDH2	1.56	1.16	2.11	0.0033674
Insulin-like Growth Factor-	IGFBP-2	0.64	0.47	0.88	0.0052053

Binding Protein 2					
Paraoxonase (PON 3)	PON3	0.66	0.49	0.9	0.008036
Tissue-type plasminogen activator	t-PA	1.63	1.13	2.35	0.0087763
Prostasin	PRSS8	1.52	1.07	2.14	0.0179974
P-selectin glycoprotein ligand 1	PSGL-1	1.49	1.07	2.07	0.0190852
Nodal modulator 1	NOMO1	1.4	1.05	1.88	0.0237439

ESM Table 3. Odds ratio (OR), 95% confidence interval (CI) and p-value for the 29 proteins associated with prevalent diabetes in the validation analysis in the main analysis. In this additional analysis, subjects with diabetic medication (n=67) were excluded. See table 2 for the abbreviations of the proteins. The proteins are given in same order as in table 2.

Protein	OR	Lower 95%CI limit	Higher 95%CI limit	p-value
CTSD	1.61	1.18	2.20	0.002
ALDH1A1	1.40	1.01	1.95	0.041
IDUA	1.97	1.28	3.03	0.002
HAO1	1.43	0.99	2.06	0.055
Gal-4	1.16	0.82	1.62	0.399
GDF-15	1.05	0.69	1.60	0.821
LPL	0.73	0.47	1.13	0.159
IL-1ra	1.34	0.91	1.98	0.139
CTSO	1.08	0.75	1.56	0.687
SIGLEC7	1.25	0.86	1.82	0.249
PAI-1	1.92	1.31	2.84	9.2e-04
CCL16	1.92	1.15	3.21	0.012
SELE	1.57	1.03	2.40	0.036
CTSZ	1.49	1.02	2.18	0.040
ACE2	1.32	0.93	1.89	0.118
VSIG2	1.31	0.90	1.90	0.159

ENPP7	1.50	1.04	2.17	0.030
TR-AP	1.53	1.04	2.24	0.031
CDH2	1.25	0.85	1.85	0.252
IGFBP-2	0.65	0.43	0.96	0.030
FABP4	1.40	0.89	2.20	0.149
DDC	1.21	0.83	1.77	0.320
PON3	0.79	0.53	1.17	0.240
IL-1RT1	1.26	0.86	1.86	0.235
t-PA	2.50	1.51	4.16	3.8e-04
KIM-1	1.18	0.77	1.79	0.446
PRSS8	1.45	0.95	2.20	0.083
PSGL-1	1.24	0.83	1.85	0.295
NOMO1	0.96	0.66	1.38	0.808

ESM Table 4. Genotypes used as instrumental variables for all proteins where single nucleotide variants located within the respective protein's gene with genome-wide significant association ($P < 5x10^{-8}$) with protein levels could be identified in EpiHealth and/or literature review. For protein abbreviations, see table 2.

**Protein**	**Original SNP**	**Proxy/availability**	**Effect allele**	**Other allele**	**beta**	**SE**	**p-value**
			**EpiHealth GWAS (SNP-exposure associations)**				
CCL16	rs11080369	NA	C	A	-1.27	0.05	3.27e-98
CTSD	rs7122341	NA	C	T	-0.33	0.03	4.93e-18
IDUA	rs3796622	NA	C	T	0.64	0.02	1.84e-97
IL-1Ra	rs4251961	NA	C	T	-0.25	0.03	1.68e-15
LPL	rs325	NA	C	T	-0.60	0.05	2.84e-32
TIM-1	rs6878069	NA	G	A	-0.52	0.02	3.72e-74
VSIG2	rs11219769	NA	T	G	0.26	0.03	4.13e-15
GDF15	rs1227731	NA	A	G	0.47	0.04	1.97e-25
IL1RT1	rs1024794	NA	G	A	0.21	0.03	1.14e-12

PON3	rs10487132	NA	G	A	0.36	0.028	1.53e-34
**Previously published GWAS (SNP-exposure associations)**							
CTSD	rs55861089	rs72850969 (only T2D)	A	G	0.41	0.03	8.0e-26
SELPLG	rs73191242	rs8179151 (only T2D)	G	A	0.20	0.03	2.0e-12
IDUA	rs3822020	rs3796622 (only FG, HOMA-IR)	G	A	0.61	0.02	7.2e-148
CATZ	rs199825459	NA	G	A	2.20	0.12	2.6e-76
ENPP7	rs11871061	available for FG, HOMA-IR, no proxy in T2D	C	T	0.99	0.02	4.9e-531
GDF15	rs45543339	rs16982345 (only FG, HOMA-IR)	T	C	0.58	0.03	1.4e-99
IL-1Ra	rs6761276	available for FG, HOMA-IR, T2D	C	T	-0.19	0.02	1.5e-14
TIM-1	rs6889164	available for FG, HOMA-IR, T2D	C	T	-0.43	0.03	2.5e-59
Siglec-7	rs140185670	NA	C	G	-0.61	0.05	1.7e-36

ESM Table 5. Mendelian randomization analysis regarding causal effects of proteins on diabetes and two other related traits, fasting glucose and the HOMA insulin resistance index. In the upper part of the table are proteins for which a significant instrumental variable (IV) was found in EpiHealth given. In the lower part, IVs from the literature were used. FG= fasting glucose, HOMA= HOMA insulin resistance index, T2D= type 2 diabetes. For protein abbreviations, see table 2.

Exposure	Outcome	IV estimate	SE	P-value
**EpiHealth GWAS (SNP-exposure associations)**				
CCL16	FG	-0.001	0.003	6.9E-01
	HOMA	0.005	0.006	4.6E-01
	T2D	-0.012	0.015	4.2E-01
CTSD	T2D	-0.012	0.029	6.8E-01
IDUA	FG	0.005	0.006	4.0E-01
	HOMA	0.000	0.007	9.8E-01
IL1RA	FG	0.018	0.013	1.6E-01
	HOMA	-0.004	0.017	8.1E-01
	T2D	0.004	0.030	8.9E-01
LPL	FG	0.014	0.008	1.1E-01
	HOMA	0.022	0.011	4.6E-02
	T2D	0.100	0.020	6.5E-07
TIM-1	FG	-0.005	0.006	3.7E-01
	HOMA	-0.010	0.007	1.8E-01
	T2D	0.024	0.015	1.2E-01
VSIG2	FG	0.013	0.013	3.3E-01
	HOMA	-0.004	0.017	8.1E-01
	T2D	0.007	0.034	8.5E-01
GDF15	FG	0.001	0.010	9.3E-01
	HOMA	0.009	0.013	5.1E-01
IL1RT1	FG	0.007	0.015	6.4E-01
	HOMA	-0.006	0.019	7.4E-01
	T2D	-0.030	0.038	4.4E-01
PON3	FG	0.014	0.009	1.2E-01

	HOMA	0.001	0.012	9.2E-01
	T2D	-0.020	0.022	3.6E-01
**Previously published GWAS (SNP-exposure associations)**				
IDUA	FG	0.005	0.006	4.0E-01
	HOMA	-1.6E-04	0.008	9.8E-01
IL1ra	FG	0.006	0.016	7.0E-01
	HOMA	-0.006	0.022	7.9E-01
	T2D	-0.015	0.043	7.3E-01
GDF15	FG	-0.016	0.007	2.3E-02
	HOMA	0.001	0.010	9.1E-01
ENPP7	FG	0.003	0.004	4.8E-01
	HOMA	0.010	0.008	2.1E-01
TIM-1	FG	-0.041	0.024	8.3E-02
	HOMA	0.002	0.031	9.5E-01
	T2D	0.101	0.057	7.3E-02
CTSD	T2D	-0.031	0.034	3.6E-01
SELPLG	T2D	0.060	0.048	2.1E-01

ESM Table 6. Mendelian randomization analyses regarding the causal effect of three glucose-related traits, diabetes (T2D), fasting glucose (FG), and insulin resistance (HOMA-IR) on protein levels. Both the inverse variance weighted and the MR Egger estimated were calculated for each pair of glucose trait and protein.

Exposure	Outcome	No. SNPs	Method	IV estimate	SE	P-value	Heterogeneity P-value	Egger intercept P-value
T2D		120	Inverse variance weighted	-0.020	0.047	6.7E-01	3.2E-01	
			MR Egger	-0.154	0.117	1.9E-01	3.3E-01	0.217
FG	ACE2	29	Inverse variance weighted	-0.159	0.216	4.6E-01	9.3E-01	
			MR Egger	-0.089	0.396	8.2E-01	9.0E-01	0.835
HOMA-IR		9	Inverse variance weighted	1.931	1.228	1.2E-01	6.3E-02	
			MR Egger	-7.244	4.552	1.6E-01	2.4E-01	0.077
T2D		120	Inverse variance weighted	0.001	0.045	9.9E-01	5.6E-01	
	CCL16		MR Egger	-0.136	0.112	2.3E-01	5.8E-01	0.187
FG		29	Inverse variance weighted	-0.138	0.212	5.1E-01	7.4E-01	

			MR Egger	0.038	0.388	9.2E-01	7.1E-01	0.593
HOMA-IR		9	Inverse variance weighted	2.284	1.058	3.1E-02	1.8E-01	
			MR Egger	-6.139	3.891	1.6E-01	4.8E-01	0.062
T2D		120	Inverse variance weighted	0.080	0.050	1.1E-01	1.2E-01	
			MR Egger	-0.102	0.124	4.1E-01	1.5E-01	0.113
FG	CTSD	29	Inverse variance weighted	-0.117	0.231	6.1E-01	3.4E-01	
			MR Egger	-0.139	0.431	7.5E-01	2.9E-01	0.950
HOMA-IR		9	Inverse variance weighted	1.670	0.920	7.0E-02	6.6E-01	
			MR Egger	0.499	4.053	9.1E-01	5.6E-01	0.775
T2D		120	Inverse variance weighted	0.042	0.050	3.9E-01	4.0E-02	
			MR Egger	-0.179	0.123	1.5E-01	6.0E-02	0.053
FG	CTSZ	29	Inverse variance weighted	-0.298	0.214	1.6E-01	4.4E-01	
			MR Egger	0.135	0.388	7.3E-01	4.8E-01	0.195
HOMA-IR		9	Inverse variance weighted	1.597	0.884	7.1E-02	9.1E-01	

			MR Egger	1.148	3.890	7.8E-01	8.6E-01	0.909
T2D		120	Inverse variance weighted	-0.088	0.051	8.3E-02	1.5E-02	
			MR Egger	-0.249	0.127	5.3E-02	1.8E-02	0.172
FG	FABP4	29	Inverse variance weighted	-0.762	0.212	3.3E-04	9.6E-01	
			MR Egger	-0.652	0.389	1.1E-01	9.5E-01	0.740
HOMA-IR		9	Inverse variance weighted	-1.370	1.204	2.5E-01	6.3E-02	
			MR Egger	-7.936	5.056	1.6E-01	1.1E-01	0.224
T2D		120	Inverse variance weighted	-0.033	0.045	4.5E-01	9.2E-01	
			MR Egger	-0.028	0.112	8.0E-01	9.1E-01	0.961
FG	GDF15	29	Inverse variance weighted	-0.406	0.231	7.9E-02	2.3E-01	
			MR Egger	-0.597	0.429	1.8E-01	2.1E-01	0.600
HOMA-IR		9	Inverse variance weighted	1.110	0.884	2.1E-01	8.9E-01	
			MR Egger	1.478	3.893	7.2E-01	8.2E-01	0.925
T2D	Gal4	120	Inverse variance weighted	-0.059	0.075	4.3E-01	5.2E-23	

			MR Egger	-0.038	0.190	8.4E-01	3.1E-23	0.902
FG		29	Inverse variance weighted	0.048	0.228	8.3E-01	2.5E-01	
			MR Egger	0.166	0.426	7.0E-01	2.1E-01	0.745
HOMA-IR		9	Inverse variance weighted	0.607	1.099	5.8E-01	1.3E-01	
			MR Egger	-4.339	4.801	4.0E-01	1.5E-01	0.325
T2D		120	Inverse variance weighted	-0.005	0.050	9.2E-01	5.8E-02	
			MR Egger	-0.303	0.123	1.5E-02	1.2E-01	0.009
FG	HAOX1	29	Inverse variance weighted	-0.180	0.263	4.9E-01	5.6E-02	
			MR Egger	0.108	0.487	8.3E-01	5.1E-02	0.487
HOMA-IR		9	Inverse variance weighted	0.224	0.904	8.0E-01	5.4E-01	
			MR Egger	-5.968	3.978	1.8E-01	7.4E-01	0.154
T2D		120	Inverse variance weighted	0.058	0.050	2.5E-01	6.1E-02	
	IDUA		MR Egger	0.112	0.126	3.8E-01	5.5E-02	0.638
FG		29	Inverse variance weighted	0.032	0.217	8.8E-01	7.8E-01	

			MR Egger	0.217	0.397	5.9E-01	7.6E-01	0.584
HOMA-IR		9	Inverse variance weighted	0.909	0.903	3.1E-01	6.5E-01	
			MR Egger	-1.435	3.973	7.3E-01	5.9E-01	0.564
T2D		120	Inverse variance weighted	-0.135	0.049	6.2E-03	4.1E-02	
			MR Egger	0.124	0.122	3.1E-01	7.4E-02	0.023
FG	IGFBP2	29	Inverse variance weighted	-0.007	0.214	9.7E-01	4.3E-01	
			MR Egger	-0.359	0.392	3.7E-01	4.3E-01	0.294
HOMA-IR		9	Inverse variance weighted	-2.919	0.882	9.4E-04	7.1E-01	
			MR Egger	0.673	3.883	8.7E-01	7.1E-01	0.374
T2D		120	Inverse variance weighted	0.016	0.044	7.2E-01	5.4E-01	
			MR Egger	0.064	0.112	5.7E-01	5.2E-01	0.641
FG	IL1RT1	29	Inverse variance weighted	0.098	0.230	6.7E-01	2.3E-01	
			MR Egger	0.054	0.430	9.0E-01	1.9E-01	0.903
HOMA-IR		9	Inverse variance weighted	0.766	0.881	3.8E-01	4.8E-01	

			MR Egger	-0.842	3.981	8.4E-01	3.9E-01	0.691
T2D		120	Inverse variance weighted	-0.058	0.051	2.5E-01	7.2E-02	
			MR Egger	-0.323	0.126	1.1E-02	1.2E-01	0.024
FG	IL1ra	29	Inverse variance weighted	-0.632	0.221	4.2E-03	8.5E-01	
			MR Egger	-0.047	0.404	9.1E-01	9.3E-01	0.097
HOMA-IR		9	Inverse variance weighted	-0.082	1.101	9.4E-01	1.8E-01	
			MR Egger	-4.082	4.941	4.4E-01	1.7E-01	0.433
T2D		120	Inverse variance weighted	-0.133	0.047	5.1E-03	2.7E-01	
			MR Egger	-0.044	0.119	7.1E-01	2.7E-01	0.421
FG	LPL	29	Inverse variance weighted	-0.397	0.244	1.0E-01	1.7E-01	
			MR Egger	-0.144	0.452	7.5E-01	1.5E-01	0.511
HOMA-IR		9	Inverse variance weighted	-2.809	0.950	3.1E-03	3.6E-01	
			MR Egger	-2.837	4.471	5.5E-01	2.7E-01	0.995
T2D	PAI	120	Inverse variance weighted	0.017	0.051	7.4E-01	1.4E-02	

			MR Egger	-0.128	0.128	3.2E-01	1.6E-02	0.219
FG		29	Inverse variance weighted	-0.571	0.212	7.0E-03	7.9E-01	
			MR Egger	-0.085	0.389	8.3E-01	8.5E-01	0.148
HOMA-IR		9	Inverse variance weighted	0.737	0.935	4.3E-01	3.5E-01	
			MR Egger	-8.579	3.893	6.3E-02	8.9E-01	0.044
T2D		120	Inverse variance weighted	-0.181	0.050	3.0E-04	3.1E-02	
			MR Egger	0.064	0.124	6.1E-01	5.4E-02	0.033
FG	PON3	29	Inverse variance weighted	0.185	0.229	4.2E-01	2.6E-01	
			MR Egger	-0.069	0.423	8.7E-01	2.4E-01	0.479
HOMA-IR		9	Inverse variance weighted	-3.731	0.884	2.5E-05	9.4E-01	
			MR Egger	-0.802	3.891	8.4E-01	9.4E-01	0.465
T2D		120	Inverse variance weighted	-0.032	0.049	5.2E-01	9.9E-02	
	PRSS8		MR Egger	-0.405	0.118	8.4E-04	2.8E-01	0.001
FG		29	Inverse variance weighted	-0.172	0.223	4.4E-01	3.7E-01	

			MR Egger	0.073	0.413	8.6E-01	3.4E-01	0.484
HOMA-IR		9	Inverse variance weighted	-0.054	0.899	9.5E-01	9.9E-01	
			MR Egger	0.111	3.957	9.8E-01	9.8E-01	0.967
T2D	PSGL1	120	Inverse variance weighted	0.027	0.046	5.5E-01	9.0E-01	
			MR Egger	-0.133	0.115	2.5E-01	9.2E-01	0.131
FG		29	Inverse variance weighted	-0.029	0.217	8.9E-01	9.5E-01	
			MR Egger	0.182	0.398	6.5E-01	9.5E-01	0.532
HOMA-IR		9	Inverse variance weighted	0.572	0.904	5.3E-01	6.9E-01	
			MR Egger	-0.927	3.978	8.2E-01	6.0E-01	0.710
T2D	SELE	120	Inverse variance weighted	0.007	0.100	9.5E-01	2.4E-65	
			MR Egger	0.077	0.252	7.6E-01	1.3E-65	0.761
FG		29	Inverse variance weighted	0.176	0.212	4.1E-01	9.3E-01	
			MR Egger	0.317	0.388	4.2E-01	9.1E-01	0.667
HOMA-IR		9	Inverse variance weighted	2.335	0.883	8.2E-03	8.2E-01	

			MR Egger	4.767	3.887	2.6E-01	7.9E-01	0.541
T2D		120	Inverse variance weighted	-0.014	0.045	7.6E-01	7.8E-01	
			MR Egger	0.042	0.115	7.2E-01	7.7E-01	0.598
FG	TIM	29	Inverse variance weighted	0.093	0.216	6.7E-01	8.8E-01	
			MR Egger	0.351	0.396	3.8E-01	8.7E-01	0.444
HOMA-IR		9	Inverse variance weighted	1.103	0.901	2.2E-01	6.3E-01	
			MR Egger	-4.535	3.967	2.9E-01	7.8E-01	0.188
T2D		120	Inverse variance weighted	0.102	0.045	2.2E-02	7.5E-01	
			MR Egger	-0.040	0.112	7.2E-01	7.7E-01	0.171
FG	TRAP	29	Inverse variance weighted	0.200	0.212	3.5E-01	7.7E-01	
			MR Egger	-0.036	0.389	9.3E-01	7.5E-01	0.476
HOMA-IR		9	Inverse variance weighted	1.711	1.272	1.8E-01	3.6E-02	
			MR Egger	-5.461	5.295	3.4E-01	7.4E-02	0.207
T2D	VSIG2	120	Inverse variance weighted	-0.026	0.046	5.7E-01	4.7E-01	

			MR Egger	-0.235	0.115	4.2E-02	5.5E-01	0.048
FG		29	Inverse variance weighted	-0.323	0.216	1.4E-01	5.3E-01	
			MR Egger	0.103	0.396	8.0E-01	5.6E-01	0.212
HOMA-IR		9	Inverse variance weighted	0.396	0.901	6.6E-01	6.4E-01	
			MR Egger	-4.580	3.967	2.9E-01	7.3E-01	0.239
T2D		120	Inverse variance weighted	0.085	0.051	9.7E-02	9.2E-03	
			MR Egger	-0.144	0.127	2.6E-01	1.6E-02	0.052
FG	tPA	29	Inverse variance weighted	-0.048	0.211	8.2E-01	8.6E-01	
			MR Egger	-0.335	0.387	4.0E-01	8.5E-01	0.386
HOMA-IR		9	Inverse variance weighted	2.293	1.065	3.1E-02	1.7E-01	
			MR Egger	-3.689	4.440	4.3E-01	2.4E-01	0.209
T2D		120	Inverse variance weighted	-0.026	0.057	6.4E-01	2.5E-03	
	ALDH1A1		MR Egger	0.278	0.139	4.8E-02	7.2E-03	0.019
FG		29	Inverse variance weighted	0.335	0.236	1.6E-01	3.4E-01	

			MR Egger	0.368	0.438	4.1E-01	2.8E-01	0.928
HOMA-IR		9	Inverse variance weighted	-2.179	0.959	2.3E-02	2.9E-01	
			MR Egger	6.527	4.452	1.9E-01	5.9E-01	0.086
T2D		120	Inverse variance weighted	0.026	0.054	6.4E-01	2.0E-02	
			MR Egger	0.103	0.136	4.5E-01	1.8E-02	0.534
FG	CDH2	29	Inverse variance weighted	-0.010	0.268	9.7E-01	9.3E-02	
			MR Egger	0.088	0.497	8.6E-01	7.2E-02	0.816
HOMA-IR		9	Inverse variance weighted	-1.206	1.076	2.6E-01	1.5E-01	
			MR Egger	9.483	4.452	7.1E-02	5.3E-01	0.044
T2D		120	Inverse variance weighted	0.022	0.052	6.7E-01	9.5E-02	
			MR Egger	0.232	0.128	7.3E-02	1.2E-01	0.077
FG	CTSO	29	Inverse variance weighted	0.009	0.300	9.8E-01	1.5E-02	
			MR Egger	-0.164	0.555	7.7E-01	1.1E-02	0.712
HOMA-IR		9	Inverse variance weighted	-1.827	0.877	3.7E-02	6.7E-01	

			MR Egger	4.873	4.455	3.1E-01	8.4E-01	0.169
T2D		120	Inverse variance weighted	0.030	0.048	5.3E-01	4.6E-01	
			MR Egger	0.077	0.120	5.2E-01	4.4E-01	0.670
FG	DDC	29	Inverse variance weighted	-0.067	0.234	7.8E-01	3.5E-01	
			MR Egger	-0.123	0.435	7.8E-01	3.0E-01	0.878
HOMA-IR		9	Inverse variance weighted	-0.701	0.875	4.2E-01	8.3E-01	
			MR Egger	6.354	4.448	2.0E-01	9.8E-01	0.150
T2D		120	Inverse variance weighted	-0.007	0.061	9.1E-01	2.9E-05	
			MR Egger	0.229	0.151	1.3E-01	5.8E-05	0.092
FG	ENPP7	29	Inverse variance weighted	-0.048	0.340	8.9E-01	6.8E-04	
			MR Egger	-0.657	0.609	2.9E-01	1.2E-03	0.245
HOMA-IR		9	Inverse variance weighted	-2.013	0.979	4.0E-02	2.6E-01	
			MR Egger	7.672	4.449	1.3E-01	6.5E-01	0.062
T2D	NOMO1	120	Inverse variance weighted	-0.027	0.056	6.3E-01	5.0E-03	

			MR Egger	0.210	0.139	1.3E-01	8.7E-03	0.065
FG		29	Inverse variance weighted	0.031	0.243	9.0E-01	2.7E-01	
			MR Egger	-0.076	0.449	8.7E-01	2.3E-01	0.778
HOMA-IR		9	Inverse variance weighted	-1.942	1.024	5.8E-02	2.1E-01	
			MR Egger	9.960	4.457	6.1E-02	8.4E-01	0.030
T2D		120	Inverse variance weighted	-0.028	0.048	5.7E-01	3.9E-01	
			MR Egger	0.186	0.120	1.2E-01	4.6E-01	0.054
FG	SIGLEC7	29	Inverse variance weighted	0.310	0.292	2.9E-01	2.5E-02	
			MR Egger	0.709	0.532	2.0E-01	2.5E-02	0.378
HOMA-IR		9	Inverse variance weighted	-0.763	0.877	3.8E-01	6.2E-01	
			MR Egger	4.882	4.456	3.1E-01	7.1E-01	0.237

ESM Figure 1. Pearson´s correlation coefficient for relationships between the 29 proteins identified as being related to diabetes.

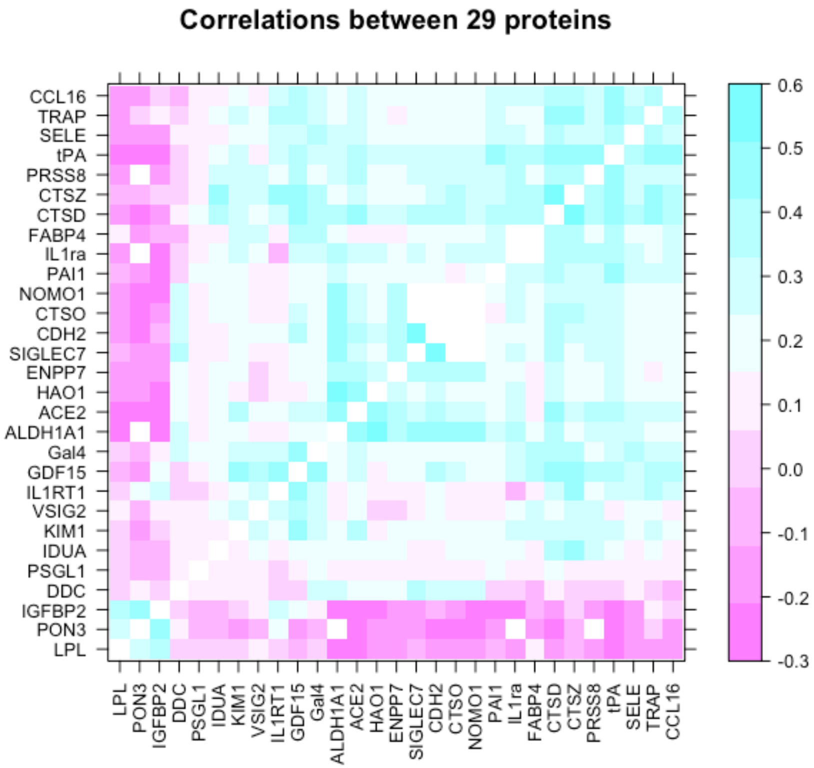

Supplement: Supplementary file 1 — (PDF 295 kb) [file 125_2019_4960_MOESM1_ESM.pdf]
